# Supplementary material for: Association of Functional Gastrointestinal Disorders with Adolescent Idiopathic Scoliosis
Source: Children (Basel). 2024 Jan 18;11(1):118. doi: 10.3390/children11010118 (PMC10814149; doi:10.3390/children11010118)
Supplement: Supplementary file 1 [file children-11-00118-s001.zip › children-2796566-supplementary.pdf]

Supplementary Materials

Table S1. Multivariable logistic regression analysis of adolescents with AIS, compared with age-matched control adolescents, in 2012

Variables	AIS (n = 2023)	Control (n = 186,757)	Adjusted Odds Ratio (95% CI)	*p* Value
FGIDs, % (n)				
Without FGID	75 (1525)	79 (147,567)		
With FGID	25 (498)	21 (39,190)	1.21 (1.10–1.35)	<.001
Gender, % (n)				
Boys	37 (746)	52 (96,668)		
Girls	63 (1277)	48 (90,089)	1.84 (1.68–2.01)	<.001
Age, % (n)				
10–12 y	23 (460)	32 (59,538)		
13–15 y	42 (845)	28 (52,838)	2.04 (1.82–2.28)	<.001
16–19 y	35 (718)	40 (74,381)	1.21 (1.08–1.37)	<.001
Insurance type, % (n)				
National health insurance	96 (1943)	96 (178,428)		
Medical aid	4 (80)	4 (8329)	0.86 (0.68–1.07)	.176
Residential district, % (n)				
Metropolitan	26 (534)	16 (30,406)		
City	31 (619)	25 (46,585)	0.76 (0.67–0.85)	<.001
Rural	43 (870)	59 (109,766)	0.45 (0.41–0.51)	<.001

Table S2. Multivariable logistic regression analysis of adolescents with AIS, compared with age-matched control adolescents, in 2013

Variables	AIS (n = 1918)	Control (n = 180,183)	Adjusted Odds Ratio (95% CI)	*p* Value
FGIDs, % (n)				
Without FGID	75 (1440)	80(144,084)		
With FGID	25 (478)	20 (36,099)	1.31 (1.18–1.46)	<.001
Gender, % (n)				
Boys	38 (729)	52 (92,945)		
Girls	62 (1189)	48 (87,238)	1.73 (1.58–1.90)	<.001
Age, % (n)				
10–12 y	22 (426)	31 (55,581)		
13–15 y	40 (774)	29 (51,359)	1.94 (1.72–2.19)	<.001
16–19 y	37 (718)	41 (73,243)	1.25 (1.11–1.41)	<.001
Insurance type, % (n)				
National health insurance	97 (1867)	96 (172,919)		
Medical aid	3 (51)	4 (7264)	0.63 (0.48–0.83)	.001
Residential district, % (n)				
Metropolitan	26 (495)	16 (29,358)		
City	27 (527)	25 (45,087)	0.70 (0.62–0.79)	<.001
Rural	47 (896)	59 (105,738)	0.51 (0.45–0.56)	<.001

Table S3. Multivariable logistic regression analysis of adolescents with AIS, compared with age-matched control adolescents, in 2014

Variables	AIS (n = 2004)	Control (n = 175,626)	Adjusted Odds Ratio (95% CI)	*p* Value
FGIDs, % (n)				
Without FGID	77 (1544)	81 (142,171)		
With FGID	23 (460)	19 (33,455)	1.24 (1.12–1.38)	<.001
Gender, % (n)				
Boys	39 (773)	52 (90,646)		
Girls	61 (1231)	48 (84,980)	1.69 (1.55–1.85)	<.001
Age, % (n)				
10–12 y	19 (376)	30 (52,166)		
13–15 y	40 (808)	29 (50,776)	2.19 (1.94–2.48)	<.001
16–19 y	41 (820)	41 (72,684)	1.54 (1.36–1.74)	<.001
Insurance type, % (n)				
National health insurance	97 (1942)	96 (168,941)		
Medical aid	3 (62)	4 (6685)	0.78 (0.61–1.01)	.057
Residential district, % (n)				
Metropolitan	26 (518)	16 (27,958)		
City	26 (528)	25 (43,577)	0.65 (0.58–0.74)	<.001
Rural	48 (958)	59 (104,091)	0.50 (0.45–0.55)	<.001

Table S4. Multivariable logistic regression analysis of adolescents with AIS, compared with age-matched control adolescents, in 2016

Variables	AIS (n = 1851)	Control (n = 163,796)	Adjusted Odds Ratio (95% CI)	*p* Value
FGIDs, % (n)				
Without FGID	76 (1413)	82 (133,579)		
With FGID	24 (438)	18 (30,217)	1.35 (1.21–1.50)	<.001
Gender, % (n)				
Boys	37 (677)	51 (84,308)		
Girls	63 (1174)	49 (79,488)	1.81 (1.65–2,00)	<.001
Age, % (n)				
10–12 y	22 (405)	30 (48,666)		
13–15 y	38 (694)	28 (45,057)	1.83 (1.60–2.05)	<.001
16–19 y	41 (752)	43 (70,073)	1.26 (1.11–1.42)	<.001
Insurance type, % (n)				
National health insurance	97 (1788)	96 (157,560)		
Medical aid	3 (63)	4 (6236)	0.86 (0.67–1.11)	.245
Residential district, % (n)				
Metropolitan	26 (482)	16 (25,472)		
City	29 (528)	24 (39,729)	0.71 (0.62–0.80)	<.001
Rural	45 (841)	60 (98,595)	0.45 (0.41–0.51)	<.001
